# Efficacy and safety of cinacalcet compared with other treatments for secondary hyperparathyroidism in patients with chronic kidney disease or end-stage renal disease: a meta-analysis

**DOI:** 10.1186/s12882-019-1639-9

**Published:** 2020-07-31

**Authors:** Yiting Sun, Binyao Tian, Zitong Sheng, Pengzhi Wan, Tianhua Xu, Li Yao

**Affiliations:** 1 Department of Clinical Medicine, China Medical University, Shenyang, 110122 Liaoning China; 2 Department of Nephrology, the First Hospital of China Medical University, Shenyang, 110001 Liaoning China

**Keywords:** Cinacalcet, Chronic kidney disease, End-stage renal disease, Meta-analysis, Secondary hyperparathyroidism

## Abstract

**Background:**

It is controversial for the effect and safety between cinacalcet and other treatments in treating secondary hyperparathyroidism for patients with chronic kidney disease (CKD) or end-stage renal disease (ESRD).

**Methods:**

Embase, PubMed, and Cochrane Library were searched through Feb 2017. 21 randomized controlled trials were included. We calculated the pooled mean difference (MD), relative risk (RR) and corresponding 95% confidence interval (CI).

**Result:**

Patients received calcimimetic agents had significantly decreased serum parathyroid hormone (MD = − 259.24 pg/mL, 95% CI: − 336.23 to − 182.25), calcium (MD = − 0.92 mg/dL, 95% CI: − 0.98 to − 0.85) and calcium phosphorus product (MD = − 5.97 mg^2^/dL^2^, 95% CI: − 9.77 to − 2.16) concentration compared with control treatment. However, the differences in cardiovascular mortality and all-cause mortality between calcimimetics agents and control group were not statistically significant. The incidence of nausea (RR = 2.13, 95% CI: 1.62 to 2.79), vomiting (RR = 1.99, 95% CI: 1.78 to 2.23) and hypocalcemia (RR = 10.10, 95% CI: 7.60 to 13.43) in CKD patients with calcimimetics agents was significantly higher than that with control treatment.

**Conclusion:**

Cinacalcet improved the biochemical parameters in CKD patients, but did not improve all-cause mortality and cardiovascular mortality. Moreover, cinacalcet can cause some adverse events.

## Background

Cardiovascular (CV) events in patients with chronic kidney disease (CKD) occur frequently [1]. CKD patient population experiences a high burden of cardiovascular mortality etyjhigher than general population. There are many causes of CV disease in chronic kidney disease, but mineral and bone disorder (MBD), including hyperphosphatemia, secondary hyperparathyroidism (SHPT) and vascular calcification. SHPT frequently occurs in patients with chronic renal failure. It is well developed before patients enters ESRD and as hemodialysis progresses, the patient’s parathyroid hormone (PTH) levels gradually increase. Traditional SHPT therapies generally includes Vitamin D sterols and phosphate binders. Although Vitamin D sterols can be effective in reducing serum intact PTH (iPTH) levels, it also increases serum levels of calcium and phosphorus, but also leading to hypercalcemia and an elevated serum calcium–phosphorus product [2, 3]. Percutaneous ethanol injection and parathyroidectomy therapies, have potential complications including throat necrosis, laryngeal recurrent nerve injury lead to weakness or paralysis of vocal cord, impaired healing, prolonged pain, and other issues related to surgical intervention.

Cinacalcet, an orally administered calcimimetic agent was originally approved in 2004 for the treatment of SHPT in patients with ESRD [4]. Cinacalcet acts by increasing the sensitivity of the calcium-sensing receptor (CaSR) on parathyroid cells to extracellular calcium ion levels, thereby decreasing serum PTH without increasing serum calcium, phosphate or the calcium phosphate product (Ca × P) in SHPT patients [4, 5]. In addition, cinacalcet lowers serum fibroblast growth factor-23 (FGF23) levels in haemodialysis patients.

The difference of effective and safety of cinacalcet in treatment of secondary hyperparathyroidism in patients with CKD or ESRD with other treatments is controversial [4, 6–25]. Therefore, we performed a meta-analysis and systematic review to quantitatively assess this relationship.

## Methods

### Search strategy

The PubMed, Cochrane Library and Embase databases were searched using the following key terms: (“cinacalcet”) AND (“chronic kidney disease/CKD” OR “end-stage renal disease/ESRD”) (last updated in Feb. 2017). Moreover, references in relevant articles were also manually cross-searched for additional trials.

### Selection criteria

The findings are reported according to the Preferred Reporting Items for Systematic Reviews and Meta-Analyses (PRISMA) [26]. All studies had to meet the following inclusion criteria: (a) study design had to be a RCT based on human subjects; (b) interventions had to be calcimimetic agents vs. control treatment; and (c) studies should report at least one of the outcomes with serum PTH, calcium, phosphate, calcium phosphorus product, all-cause mortality, nausea, vomiting, cardiovascular mortality, hypercalcemia and hypocalcemia. The following exclusion criteria were used: (a) abstracts or overlapped studies; and (b) studies published in languages other than English; (c) studies in animal models.

### Data extraction and quality assessment

All the available data on outcomes were independently extracted by two investigators from each study based on the inclusion criteria listed above. Any disagreement was resolved by discussing with the third expert. The study characteristics were recorded as follows: first author name, publication year, country where the research was performed, number of patients, mean age, intervention method, duration of the trial, serum PTH, calcium, phosphate, calcium phosphorus product, all-cause mortality, nausea, vomiting, cardiovascular mortality, hypercalcemia and hypocalcemia. We evaluate the quality of RCTs with the Cochrane Collaboration’s tool for assessing risk of bias [27]. The assessment will include the following components: random sequence generation, blinding of patients and study personnel, allocation concealment, blinding of outcome assessment, selective reporting of outcomes, completeness of outcome data, and other threats. The following response options were included: “probably yes”, “definitely yes”, “definitely no”, and “probably no”, with “probably yes” or “definitely yes” assigned low risk of bias and “probably no” or “definitely no” assigned high risk of bias.

### Statistical analysis

All results summarized using STATA Software (version 12, Stata Corporation, College Station, TX). For the continuous data, we calculated the mean difference (MD) and 95% confidence intervals (CI). For dichotomous data, the risk ratio (RR) and 95% confidence intervals were calculated. Between-study heterogeneity was examined using χ^2^ test and *I*^*2*^, which assumes the presence of heterogeneity at *I*^*2*^ > 50%. Preliminary analysis using a fixed effect model (Mantel-Haenszel method), if there are study heterogeneity (*P* < 0.1 and/or *I*^*2*^ > 50%), using a random effects model. In the sensitivity analysis, the influence of each study on the summary effect was analyzed by dropping one study at a time. By funnel plot, Begg’s and Egger’s test to assess publication bias visually evaluated symmetry (*P* < 0.05 was considered statistically significant).

## Results

### Characteristics of the studies

**Fig. 1:**
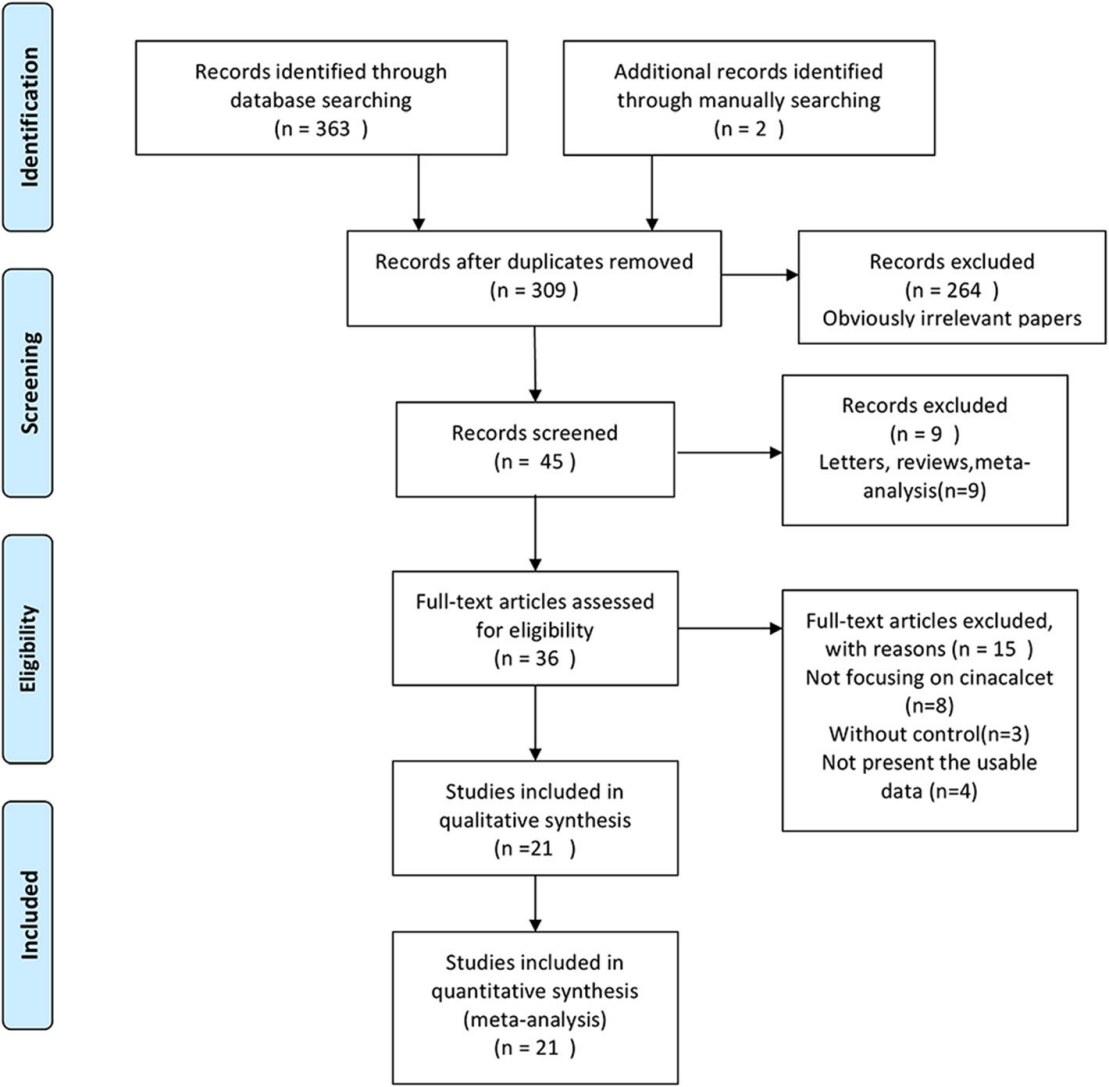
Flow diagram of studies identification

**Fig. 2:**
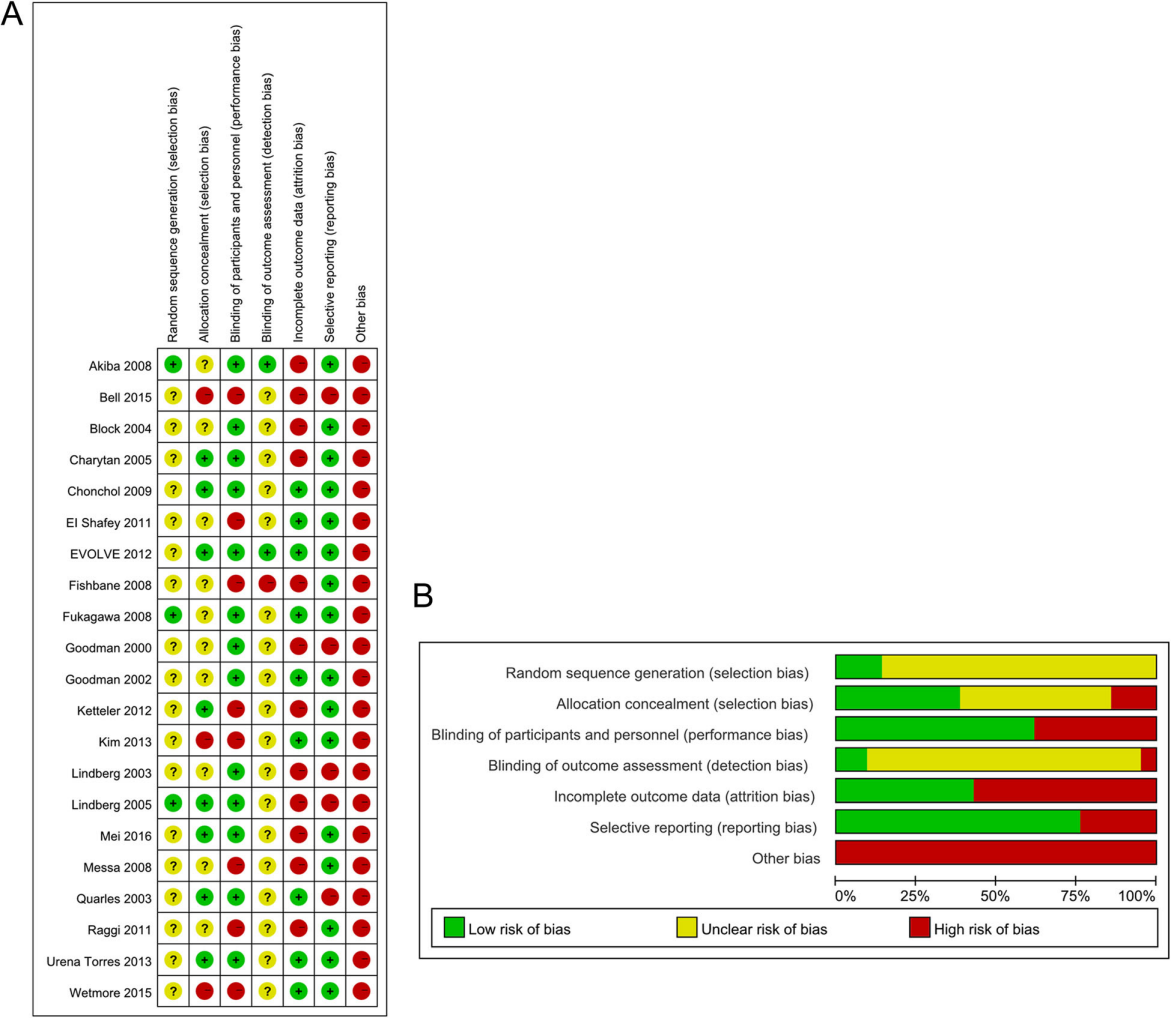
Risk of bias assessments for the randomized trials included in the meta-analysis. **A**) Risk of bias summary; **B**) Risk of bias graph. *Symbols*. (+): low risk of bias; (?): unclear risk of bias; (−): high risk of bias

As is demonstrated in Fig. 1, a total of 309 articles were identified, 273 of which were determined to be irrelevant based on review of titles and abstracts. Thus, a total of 36 full-text articles were assessed for eligibility. Of these 36 articles, 15 were excluded because them didn’t meet the inclusion criteria, including eight not focusing on cinacalcet, three without control, four not present the usable data. In total, 21 articles fulfilled the inclusion criteria and were enrolled. Of the 21 retrieved articles, 8373 participants are represented. In total, the 21 RCTs represented 4543 and 3830 patients in the calcimimetic agents and control treatment groups, respectively. The characteristics of the retrieved trials and the recorded outcomes are reported in Table 1. The treatment duration ranged from 2 to 52 weeks. A summary of selection bias, detection bias, performance bias, reporting bias, attrition bias, and other bias identified in each individual RCT is shown in Fig. 2. All of the included studies showed moderate and high quality with acceptable and moderate risk of bias.

**Table 1:** Characteristics of randomized controlled trials included in this meta-analysis

Authors/year of publication	Country	Mean age (years)	Stage of CKD	Intervention		Duration of the trial	Outcomes assessed
				Cinacalcet	Control		
Goodman/2000 [12]	USA	Cinacalcet 48.6 (12.4) Control 54.7 (16.8)	HD	R-568, 100 mg/d, *N* = 16	Placebo, *N* = 5	24 days	All-cause mortality, cardiovascular mortality, nausea, and hypocalcemia.
Goodman/2002 [13]	USA	NA	HD	AMG 073, 10–50 mg/d, *N* = 23	Placebo, *N* = 7	15 days	All-cause mortality, cardiovascular mortality, nausea, and hypocalcemia.
Lindberg/2003 [14]	USA	Cinacalcet 52.7 (16.4) Control 48.8 (15.6)	HD	AMG 073, 10–50 mg/d, *N* = 39	Placebo, N = 39	18 weeks	Serum PTH, all-cause mortality, cardiovascular mortality, nausea, and hypocalcemia.
Quarles/2003 [15]	USA	Cinacalcet 49.6 (8.5) Control 47.9 (14.2)	HD	AMG 073, 25–100 mg/d, *N* = 36	Placebo, *N* = 35	18 weeks	Serum PTH, calcium, phosphate, and calcium phosphorus product.
Block/2004 [9]	USA	Cinacalcet 54 (14) Control 55 (15)	HD	Cinacalcet, 30–180 mg/d, *N* = 371	Placebo, *N* = 370	26 weeks	Serum PTH, phosphate, calcium phosphorus product, all-cause mortality, nausea, vomiting, and hypocalcemia.
Charytan/2005 [16]	USA	Cinacalcet 60.6 (15.6) Control 61.9 (15.1)	CKD	Cinacalcet, 30–180 mg/d, *N* = 27	Placebo, *N* = 27	18 weeks	All-cause mortality, cardiovascular mortality, nausea, and hypocalcemia.
Lindberg/2005 [17]	USA	Cinacalcet 51.8 (14) Control 53.5 (13.9)	HD PD	Cinacalcet, 30–180 mg/d, *N* = 294	Placebo, *N* = 101	26 weeks	Serum PTH, calcium, phosphate, calcium phosphorus product, all-cause mortality, nausea, and vomiting.
Akiba/2008 [18]	Japan	Cinacalcet 56.7 (9.2) Control 51.8 (7.5)	HD	Cinacalcet, 12.5–50 mg/d, *N* = 91	Placebo, *N* = 30	5 weeks	All-cause mortality, cardiovascular mortality, nausea, vomiting, and hypocalcemia.
Fishbane/2008 [19]	USA	Cinacalcet 57.7 (14.9) Control 59 (12.4)	HD	Cinacalcet, 30–180 mg/d plus paricalcitol 2 g or doxercalciferol 1 g, *N* = 87	Paricalcitol 2 g or doxercalciferol 1 g, *N* = 86	27 weeks	All-cause mortality, nausea, vomiting, hypercalcemia, and hypocalcemia.
Fukagawa/2008 [20]	Japan	Cinacalcet 54.7 (11) Control 55.7 (11.7)	HD	Cinacalcet, 30–180 mg/d, *N* = 72	Placebo, *N* = 71	14 weeks	Serum calcium, phosphate, calcium phosphorus product, all-cause mortality, cardiovascular mortality, nausea, vomiting, and hypocalcemia.
Messa/2008 [21]	Italy	Cinacalcet 58.5 (14.5) Control 58.3 (14.5)	HD	Cinacalcet, 30–180 mg/d, *N* = 368	Conventional Care, *N* = 184	23 weeks	Serum PTH, calcium, phosphate, calcium phosphorus product, all-cause mortality, cardiovascular mortality, nausea, vomiting, and hypocalcemia.
Chonchol/2009 [22]	USA	Cinacalcet 64.7 (13.3) Control 66.2 (12.2)	CKD	Cinacalcet, 30–180 mg/d, *N* = 302	Placebo, *N* = 102	32 weeks	Serum calcium, phosphate, calcium phosphorus product, all-cause mortality, cardiovascular mortality, nausea, vomiting, and hypocalcemia.
EI-Shafey/2011 [23]	Egypt	Cinacalcet 51.5 (12.7) Control 51.8 (15.0)	HD	Cinacalcet, 30–180 mg/d, *N* = 55	conventional therapy (intravenous alfacalcidol thrice weekly at the end of their dialysis session and phosphate binders), N = 27	36 weeks	Serum PTH, calcium, phosphate, calcium phosphorus product, all-cause mortality, nausea, vomiting, and hypocalcemia.
Raggi/2011 [24]	USA	Cinacalcet 61.2 (12.6) Control 61.8 (12.8)	HD	Cinacalcet, 30–180 mg/d plus low-dose vitamin D, *N* = 180	Same dose of vitamin D prescribed N = 180	52 weeks	All-cause mortality, hypercalcemia, and hypocalcemia.
EVOLVE/2012 [25]	USA	Cinacalcet 55.0 (35–74) Control 54.0 (35–73)	HD	Cinacalcet, 30–180 mg/d, *N* = 1948	Placebo, *N* = 1935	20 weeks	All-cause mortality, cardiovascular mortality, nausea, vomiting, hypercalcemia, and hypocalcemia.
Ketteler/2012 [26]	Germany	Cinacalcet 59.9 (12.0) Control 61.2 (12.7)	HD	Cinacalcet (dose unclear) plus low-dose vitamin D *N* = 134	Paricalcitol 0.07 μg/kg IV or iPTH/60 PO N = 134	28 weeks	All-cause mortality, cardiovascular mortality, nausea, vomiting, hypercalcemia, and hypocalcemia.
Kim/2013 [27]	Korea	Cinacalcet 48.8 (11.5) Control 47.2 (8.4)	PD	Cinacalcet, 25–50 mg/d plus low-dose vitamin D, *N* = 33 N = 33	Same dose of vitamin D prescribed N = 33	20 weeks	All-cause mortality and cardiovascular mortality.
Urena-Torres/2013 [28]	France	Cinacalcet 57.9 (13.6) Control 57.0 (14.6)	HD	Cinacalcet, 25–50 mg/d plus low-dose vitamin D, *N* = 154	Same dose of vitamin D prescribed *N* = 155	52 weeks	All-cause mortality, nausea, vomiting, hypercalcemia, and hypocalcemia.
Bell/2015 [29]	USA	Cinacalcet 52.4 (13.7) Control 50.7 (13.6)	HD	Cinacalcet, 5–10 mg/d, *N* = 40	Placebo, *N* = 38	4 weeks	All-cause mortality, nausea, vomiting, and hypercalcemia.
Wetmore/2015[30]	USA	Cinacalcet 53 (21–81) Control 55 (22–86)	HD	Cinacalcet, 30–180 mg/d, N = 155	vitamin D (dose unclear), *N* = 157	52 weeks	Serum PTH, calcium, phosphate, all-cause mortality, hypercalcemia, and hypocalcemia.
Mei/2016[31]	China	Cinacalcet 50.02 (11.17) Control 50.12 (11.34)	HD	Cinacalcet, 25–100 mg/d, *N* = 118	Placebo, *N* = 114	16 weeks	Serum PTH, calcium, phosphate, calcium phosphorus product, nausea, vomiting, and hypocalcemia.

*CKD* Chronic kidney disease, *HD* hemodialysis, *NA* not available, *PTH* parathyroid hormone,^a^*PD* peritoneal dialysis

### Quantitative synthesis

**Fig. 3:**
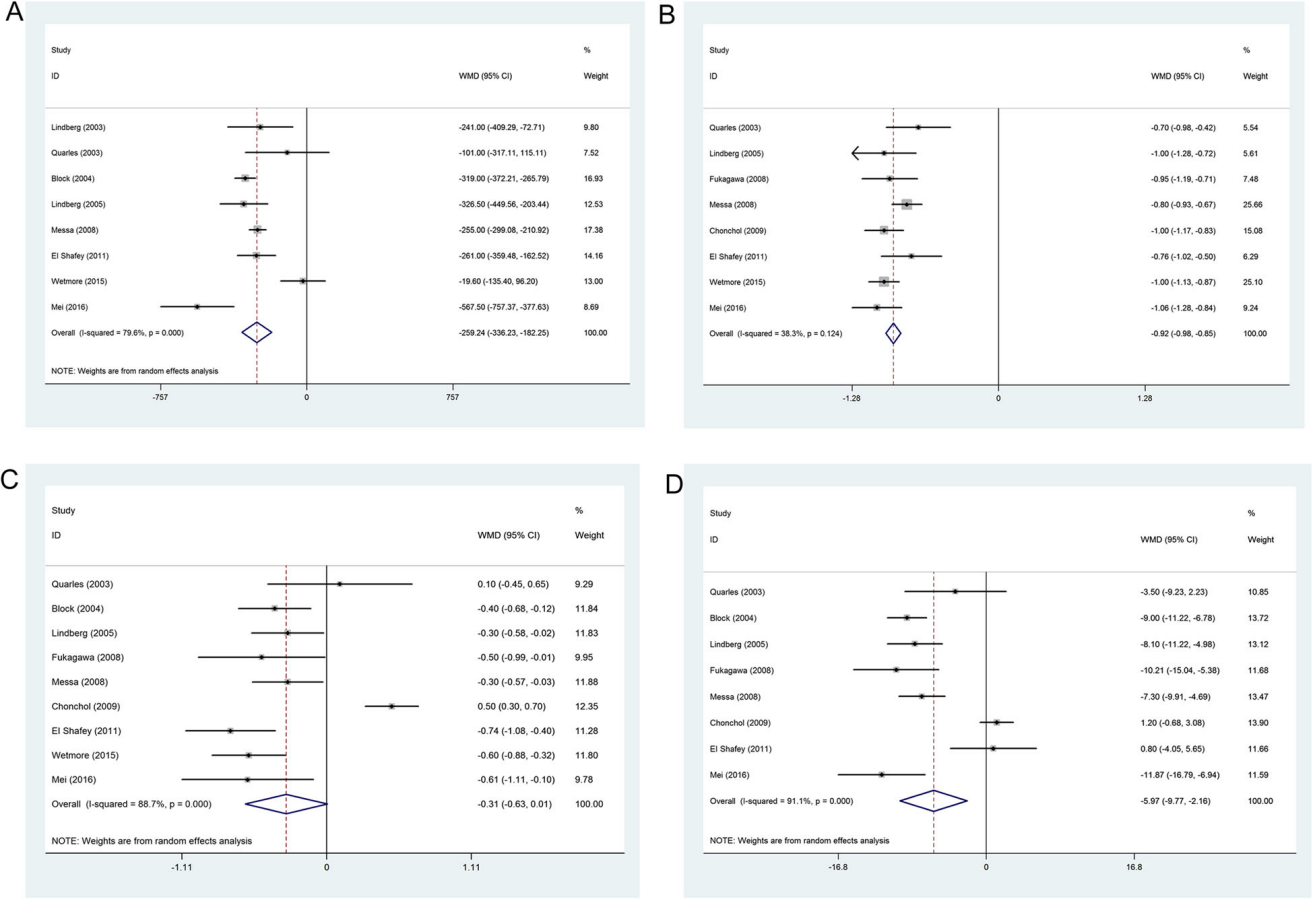
Effect of cinacalcet versus control treatment in patients with chronic kidney disease. **A**) Serum parathyroid hormone; **B**) Serum calcium; **C**) Serum phosphate; **D**) Calcium phosphorus product

The eight studies [4, 8, 9, 11, 15, 17, 24, 25] provided numerical data regarding the serum PTH concentration in patients who received calcimimetic agents and control treatment, and were included in the meta-analysis. There was evidence of heterogeneity among the 8 studies, therefore, a random-effects model of analysis was used. The pooled difference in means indicated that patients who received calcimimetic agents (MD = − 259.24 pg/mL, 95% CI: − 336.23 to − 182.25, *P*_heterogeneity_ < 0.001, *I*^*2*^ = 79.6%) had significantly decreased serum PTH concentration compared with patients who received control treatment (Fig.3a). We probed into detailed results in subgroup analyses stratified by country (USA or other country), patient median age (patient median age < 55 or ≥ 55), sample size (sample size < 200 or ≥ 200), dialysis or not and duration of the trial (duration of the trial <24w or ≥ 24w). All subgroup results were quite consistent with the overall results. The results are summarized in Table 2.

**Table 2:** Subgroup analysis of the meta-analysis

Outcomes	Subgroup	Number of trials	Effect (95% CI)	Estimate for overall effect	Heterogeneity
Serum PTH concentration	USA	5	− 209.57 (− 341.06, − 78.07)	*P* = 0.002	*I*^2^ = 83.6%, *P* < 0.001
	Other country	3	−327.13 (− 454.5, − 199.77)	*P* < 0.001	*I*^2^ = 79.8%, *P* = 0.007
	Patient median age <55	5	− 300.73 (− 417.8, − 183.67)	*P* < 0.001	*I*^2^ = 67%, *P* = 0.017
	Patient median age ≥55	3	− 211.64 (− 330.57, − 92.72)	*P* < 0.001	*I*^2^ = 90.6%, *P* < 0.001
	Sample size <200	3	− 235.15 (− 314.24, − 156.05)	*P* < 0.001	*I*^2^ = 0, *P* = 0.417
	Sample size ≥200	5	− 281.23 (− 385.59, − 176.88)	*P* < 0.001	*I*^2^ = 87.5%, *P* < 0.001
	Duration of the trial <24 w	4	− 289.97 (− 435.79, − 144.15)	*P* < 0.001	*I*^2^ = 75.5%, *P* = 0.007
	Duration of the trial ≥24 w	4	− 235.23 (− 361.52, − 108.95)	*P* < 0.001	*I*^2^ = 86.4%, *P* < 0.001
Serum phosphate concentration	USA	5	−0.14 (− 0.61, 0.32)	*P* = 0.544	*I*^2^ = 92.3%, *P* < 0.001
	Other country	4	−0.51 (− 0.73, − 0.29)	*P* < 0.001	*I*^2^ = 27.1%, *P* = 0.249
	Patient median age <55	4	−0.41 (− 0.74, − 0.09)	*P* = 0.013	*I*^2^ = 62.2%, *P* = 0.047
	Patient median age ≥55	5	−0.25 (− 0.71, 0.22)	*P* = 0.297	*I*^2^ = 92.7%, *P* < 0.001
	Sample size <200	3	− 0.42 (− 0.89, 0.05)	*P* = 0.081	*I*^2^ = 68.6%, *P* = 0.042
	Sample size ≥200	6	−0.27 (− 0.66, 0.12)	*P* = 0.175	*I*^2^ = 91.3%, *P* < 0.001
	Duration of the trial <24 w	4	− 0.33 (− 0.58, − 0.09)	*P* = 0.008	*I*^2^ = 24.9%, *P* = 0.262
	Duration of the trial ≥24 w	5	−0.30 (− 0.79, 0.19)	*P* = 0.226	*I*^2^ = 93.8%, *P* < 0.001
	Receiving dialysis	8	−0.43 (− 0.58, 0.29)	*P* < 0.001	*I*^2^ = 32.3%, *P* = 0.170
	Not receiving dialysis	1	0.50 (0.3, 0.7)	*P* < 0.001	*–*
Serum calcium phosphorus product	USA	4	−4.86 (−10.82, 1.09)	*P* = 0.109	*I*^2^ = 94.6%, *P* < 0.001
	Other country	4	−7.15 (−11.88, − 2.41)	*P* = 0.003	*I*^2^ = 80.5%, *P* = 0.002
	Patient median age <55	4	−5.79 (− 10.91, − 0.66)	*P* = 0.027	*I*^2^ = 80.4%, *P* = 0.002
	Patient median age ≥55	4	−6.18 (− 11.98, − 0.37)	*P* = 0.037	*I*^2^ = 95.1%, *P* < 0.001
	Sample size <200	3	- 4.33 (− 10.96, 2.31)	*P* = 0.201	*I*^2^ = 80.1%, *P* = 0.007
	Sample size ≥200	5	− 6.84 (− 11.77, − 1.91)	*P* = 0.007	*I*^2^ = 94.2%, *P* < 0.001
	duration of the trial <24 w	4	−8.26 (− 11.28, − 5.24)	*P* < 0.001	*I*^2^ = 48.4%, *P* = 0.121
	duration of the trial ≥24 w	4	−3.87 (−9.85, 2.12)	*P* = 0.206	*I*^2^ = 94.9%, *P* < 0.001
	Receiving dialysis	7	−7.29 (−9.77, − 4.81)	*P* < 0.001	*I*^2^ = 68.6%, *P* = 0.004
	Not receiving dialysis	1	1.20 (− 0.68, 3.08)	*P* = 0.210	*–*

*CKD* Chronic kidney disease, *HD* hemodialysis, *NA* not available, *PTH* parathyroid hormone, *PD* peritoneal dialysis

The eight studies [9, 11, 14–17, 24, 25] provided numerical data regarding the serum calcium concentration in patients who received calcimimetic agents and control treatment, and were included in the meta-analysis. There was no evidence of heterogeneity among the 8 studies, therefore, a fixed-effects model of analysis was used. The pooled difference in means indicated that patients who received calcimimetic agents (MD = − 0.92 mg/dL, 95% CI: − 0.98 to − 0.85, *P*_heterogeneity_ = 0.124, *I*^*2*^ = 38.3%) had significantly decreased serum calcium concentration compared with patients who received control treatment (Fig. 3b).

The nine studies [4, 9, 11, 14–17, 24, 25] provided numerical data regarding the serum phosphate concentration in patients who received calcimimetic agents and control treatment, and were included in the meta-analysis. There was evidence of heterogeneity among the 9 studies, therefore, a random-effects model of analysis was used. The pooled difference in means indicated that patients who received calcimimetic agents (MD = − 0.31 mg/dL, 95% CI: − 0.63 to 0.01, *P*_heterogeneity_ < 0.001, *I*^*2*^ = 88.7%) had not significantly decreased serum phosphate concentration compared with patients who received control treatment (Fig. 3c). We probed into detailed results in subgroup analyses stratified by country, patient median age, sample size, and duration of the trial. All subgroup results are summarized in Table 2.

The eight studies [4, 9, 11, 14–17, 24, 25] provided numerical data regarding the serum calcium phosphorus product in patients who received calcimimetic agents and control treatment, and were included in the meta-analysis. There was evidence of heterogeneity among the 8 studies, therefore, a random-effects model of analysis was used. The pooled difference in means indicated that patients who received calcimimetic agents (MD = − 5.97 mg^2^/dL^2^, 95% CI: − 9.77 to − 2.16, *P*_heterogeneity_ < 0.001, *I*^*2*^ = 91.1%) had significantly decreased serum calcium phosphorus product compared with patients who received control treatment (Fig. 3d). We probed into detailed results in subgroup analyses stratified by country, patient median age, sample size, and duration of the trial. All subgroup results are summarized in Table 2.

### All-cause mortality (ACM)

**Fig. 4:**
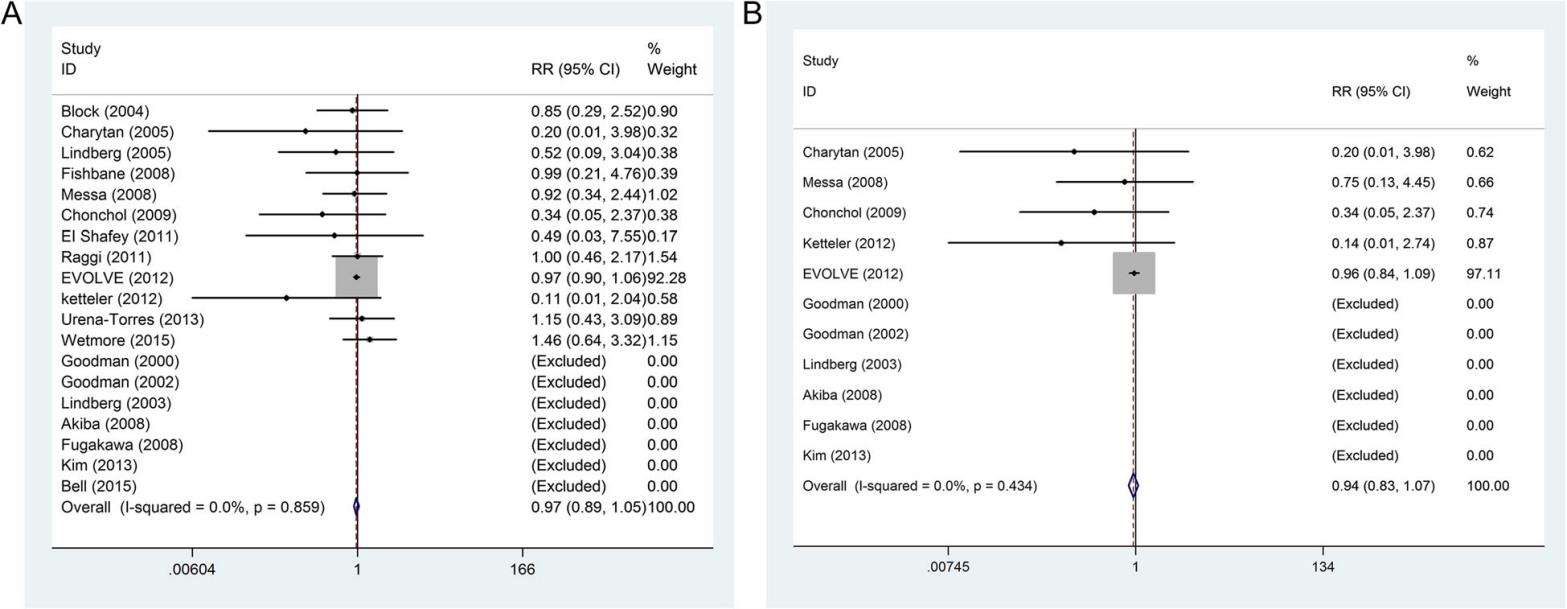
Forest plot of mortality with cinacalcet versus control treatment in patients with chronic kidney disease. **A**) All-cause mortality; **B**) Cardiovascular mortality

This outcome was reported in 19 trials. There was no significant heterogeneity between the study (*P* = 0.859, *I*^*2*^ = 0%), the fixed effect model was used. There was no significant difference in the incidence of ACM in patients received calcimimetic agents compared with control treatment group (RR = 0.97, 95% CI: 0.89 to 1.05), as shown in Fig. 4a.

### Cardiovascular mortality (CVM)

This outcome was reported in eleven trials. There was no significant heterogeneity between the study (*P* = 0.434, *I*^*2*^ = 0%), the fixed effect model was used. There was no significant difference in the incidence of CVM (RR = 0.94, 95% CI: 0.83 to 1.07), as shown in Fig. 4b.

The nineteen studies [4, 6–8, 10–17] were included in the meta-analysis of adverse events.

### Nausea

**Fig. 5:**
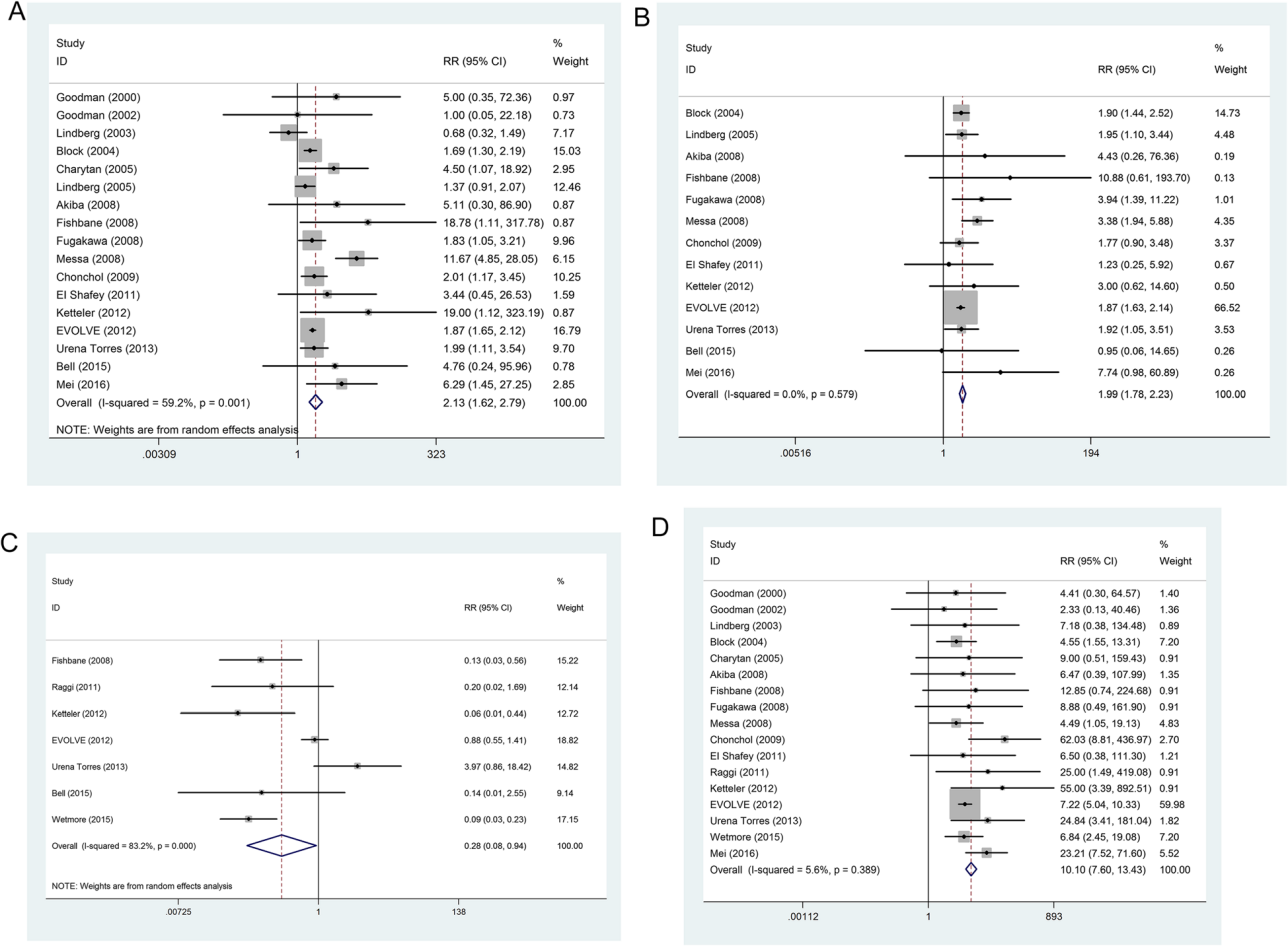
Pooled risk ratio of adverse events with cinacalcet versus control treatment in patients with chronic kidney disease. **A**) Nausea; **B**) Vomiting; **C**) Hypercalcemia; **D**) Hypocalcemia

This outcome was reported in 17 trials. There was significant heterogeneity between the study (*P* = 0.001, *I*^*2*^ = 59.2%), the random effect model was used. There was significantly increased the incidence of nausea in patients received calcimimetic agents compared with control treatment group (RR = 2.13, 95% CI: 1.62~2.79), as shown in Fig. 5a.

### Vomiting

This outcome was reported in 13 trials. There was no significant heterogeneity between the study (*P* = 0.579, *I*^*2*^ = 0%), the fixed effect model was used. There was significantly increased the incidence of vomiting in patients received calcimimetic agents compared with control treatment group (RR = 1.99, 95% CI: 1.78 to 2.23), as shown in Fig. 5b.

### Hypercalcemia

This outcome was reported in seven trials. There was significant heterogeneity between the study (*P* < 0.001, *I*^*2*^ = 83.2%), the random effect model was used. There was significantly decreased the incidence of hypercalcemia in patients received calcimimetic agents compared with control treatment group (RR = 0.28, 95% CI: 0.08 to 0.94), as shown in Fig. 5c.

### Hypocalcemia

This outcome was reported in 17 trials. There was no significant heterogeneity between the studies (*P* = 0.389, *I*^*2*^ = 5.6%), the fixed effect model was used. There was significantly increased the incidence of hypocalcemia in patients received calcimimetic agents compared with control treatment group (RR = 10.10, 95% CI: 7.60 to 13.43), as shown in Fig. S1D.

### Sensitivity analysis

We performed sensitivity analyses to assess the stability of the results by sequential removing each study. Any single study was removed, while the overall statistical results do not change, indicating that the results of this study are statistically robust.

### Publication bias

**Fig. 6:**
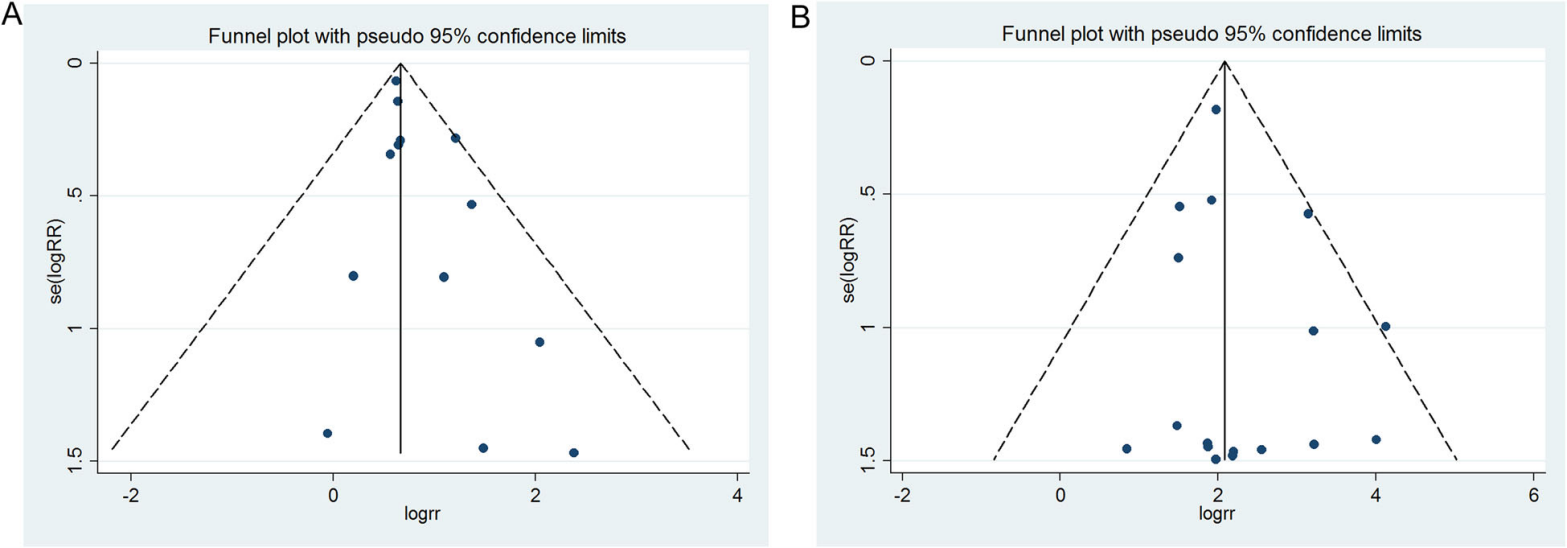
Funnel plot for publication bias test. Each point represents a separate study for the indicated association. (**A**) Incidence of vomiting; and (**B**) incidence of hypocalcemia

Egger’s, Begg’s test and funnel plot were performed to evaluate publication bias of the literatures. Funnel plots revealed no evidence of publication bias for incidence of vomiting (Begg’s test *P* = 0.428; Egger’s test *P* = 0.063) (Fig. 6a) and incidence of hypocalcemia (Begg’s test *P* = 0.592; Egger’s test *P* = 0.251) (Fig. 6b).

## Discussion

A comprehensive search was conducted, and finally 21 randomized clinical trials involving 8373 CKD patients met our inclusion criteria. Our study showed that patients who received calcimimetic agents had significantly decreased serum PTH (MD = − 259.24 pg/mL, 95% CI: − 336.23 to − 182.25, *P*_heterogeneity_ < 0.001, *I*^*2*^ = 79.6%), calcium (MD = − 0.92 mg/dL, 95% CI: − 0.98 to − 0.85, *P*_heterogeneity_ = 0.124, *I*^*2*^ = 38.3%) and calcium phosphorus product (MD = − 5.97 mg^2^/dL^2^, 95% CI: − 9.77 to − 2.16, *P*_heterogeneity_ < 0.001, *I*^*2*^ = 91.1%) concentration compared with patients who received control treatment. However, there was no significant difference in cardiovascular mortality and all-cause mortality between calcimimetics agents and control treatment group. Furthermore, we observed that the incidence of adverse events (nausea, vomiting and hypocalcemia) in CKD patients treated with calcimimetics agents was significantly higher than that with control treatment.

The efficacy and safety of calcimimetic agents have been investigated by several meta-analyses. As far as we know, this meta-analysis is the largest one to evaluate the efficacy and safety profile of calcimimetic agents up to now, which involved 8373 CKD patients from 21 RCTs. Recently, Palmer et al. [28] performed a meta-analysis about the efficacy and safety of cinacalcet in CKD patients. Compared with Palmer’s work, we identified more eligible studies. Compared with another meta-analysis about calcimimetic agents reported by Sekercioglu et al. [29], we included more new RCTs, involved more CKD patients and performed a detailed analysis. Vascular calcification is a very common and serious problem in adult ESRD patients and is significantly associated with cardiovascular disease and mortality. In this study, there was no evidence of a reduction in cardiovascular mortality and all-cause mortality in cinacalcet compared with the control. These results are consistent with previous findings. Heterogeneity is a problem with most meta-analyses. In this meta-analysis, heterogeneity was found in the subgroup and overall analyses; thus, we used the random-effects model. Based on the data collected, we suggested that the sample size and duration of the trial have at least partly contributed to the between-study heterogeneity. However, clinical heterogeneity may or may not produce detectable statistical heterogeneity. Important clinical differences between studies, for example, between patient populations, intervention protocols and the types and timing of study outcomes - can be present in the absence of statistical heterogeneity. These differences can limit the appropriateness of statistical synthesis of individual study estimates through meta-analysis. Furthermore, we performed sensitivity analyses to assess the stability of the results by sequential removing each study. Any single study was removed, while the overall statistical results do not change, indicating that the results of this study are statistically robust.

SHPT and MBD are common in many patients with CKD. Due to increased risk of CVD, occurrence of fractures and mortality, two clinical conditions bring a large amount medical burden. Management of SHPT and MBD in patients with CKD is made by controlling the triggering factors. Sometimes these measures are not effective, and maybe even have adverse effects. As the main physiological factors of the two entities, the calcium agent provides a therapeutic advantage. Firstly, they reduce the level of serum PTH by inhibiting their secretion, and secondly, they stimulate the synthesis of the vitamin D receptor in the parathyroid gland, which increases the sensitivity to vitamin D and subsequently inhibits PTH. At present, focus has moved to the effect of cinacalcet on hard clinical end point, and ADVANCE [18] and EVOLVE [19], two randomized controlled trials, have been performed to assess the impact of cinacalcet on CV calcification and the risk of CV events and mortality. Although the initial analysis of the two trials did not reveal significant effects of cinacalcet, the advantage of cinacalcet was proposed to analyze the potential problems considered in the test. These positive results and experimental studies showed that the good effects of cinacalcet on bone metabolism and vascular calcification.

Meanwhile, some limitations should be noticed in this meta-analysis: First, there was a significant heterogeneity. Selection criteria for different patients and control treatment options are possible explanations for heterogeneity. Second, language can also produce a bias. Specifically, we only choose English or exclude other qualified research. Third, several studies of small sample sizes, may reduce the statistical power. Finally, our results were based on unadjusted assessment of RRs, which might influence the results. Based on these limitations, the results should be considered carefully.

## Conclusion

In conclusion, despite the limitations of this meta-analysis, our study confirmed that patients who received calcimimetic agents had significantly decreased serum PTH, calcium and calcium phosphorus product compared with patients who received control treatment. However, there was no significant difference in all-cause mortality and cardiovascular mortality between calcimimetics agents and control treatment group. Further studies with larger data set and well-designed models are required to validate our findings.

## Data Availability

The datasets used and/or analyzed during the current study are available from the corresponding author on reasonable request.

## Abbreviation

ACM: All-cause mortality
CaSR: Calcium-sensing receptor
CI: Confidence interval
CKD: Chronic kidney disease
CV: Cardiovascular
CVM: Cardiovascular mortality
ESRD: End-stage renal disease
iPTH: Intact PTH
MBD: Mineral and bone disorder
MD: Mean difference
PTH: Parathyroid hormone
RR: Relative risk
SHPT: Secondary hyperparathyroidism
